# Prediabetes increases the risk of major limb and cardiovascular events

**DOI:** 10.1186/s12933-023-02085-y

**Published:** 2023-12-19

**Authors:** Jung-Chi Hsu, Yen-Yun Yang, Shu-Lin Chuang, Jen-Kuang Lee, Lian-Yu Lin

**Affiliations:** 1 Division of Cardiology, Department of Internal Medicine, National Taiwan University Jinshan Branch, New Taipei City, Taiwan; 2 Division of Cardiology, Department of Internal Medicine, National Taiwan University College of Medicine and Hospital, No. 7, Chung-Shan South Road, Taipei, 100 Taiwan; 3 Department of Medical Research, National Taiwan University Hospital, Taipei, Taiwan; 4 Department of Laboratory Medicine, National Taiwan University College of Medicine, Taipei, Taiwan; 5 Cardiovascular Center, National Taiwan University Hospital, Taipei, Taiwan; 6 Telehealth Center, National Taiwan University Hospital, Taipei, Taiwan; 7 Department of Internal Medicine, College of Medicine, National Taiwan University, Taipei, Taiwan; 8 Master’s Program in Smart Medicine and Health Informatics, NTU, Taipei, 10617 Taiwan

**Keywords:** Prediabetes, Peripheral arterial Disease, Major adverse limb event (MALE), Major cardiovascular event (MACE)

## Abstract

**Background:**

Prediabetes, an intermediate stage between normal blood sugar levels and a diabetes mellitus diagnosis, is increasing in prevalence. Severe prediabetes is associated with a similar risk of complications as diabetes, but its relationship with peripheral arterial disease remains underexplored.

**Methods:**

We conducted a retrospective cohort study involving 36,950 adult patients, utilizing electronic medical records from the National Taiwan University Hospital between 2014 and 2019. We employed multivariable Cox regression and Kaplan–Meier analysis with the log-rank test to analyze major adverse limb events (MALE) and major adverse cardiovascular events (MACE) in relation to normal glucose regulation (NGR) and prediabetes.

**Results:**

During the 131,783 person-years follow-up, 17,754 cases of prediabetes and 19,196 individuals with normal glucose regulation (NGR) were identified. Kaplan–Meier analysis revealed an increased incidence of both MALE and MACE in individuals with prediabetes. (log-rank *p* = 0.024 and < 0.001). Prediabetes exhibited a significant association with an elevated risk of MALE (adjusted hazard ratio (aHR) 1.26 [95% CI 1.10–1.46], *p* = 0.001) and MACE (aHR 1.46 [1.27–1.67], *p* < 0.001). Furthermore, in individuals with prediabetes, the elevation in the risk of MALE commenced before HbA1c levels surpassed 5.0% (for HbA1c 5.0-5.5%: aHR 1.78 (1.04–3.04), *p* = 0.036; HbA1c 5.5-6.0%: aHR 1.29 [1.06–1.58], *p* = 0.012; aHbA1c 6.0-6.5%: aHR 1.39 [1.14–1.70], *p* < 0.001). Similarly, the onset of increased MACE risk was observed when HbA1c levels exceeded 5.5% (for HbA1c 5.5–6.0%: aHR 1.67 [1.39–2.01], *p* < 0.001; HbA1c 6.0-6.5%: HR 2.10 [1.76–2.51], *p* < 0.001). Factors associated with both MALE and MACE in prediabetes include advanced age, male gender, higher body mass index, and a history of heart failure or atrial fibrillation.

**Conclusion:**

We demonstrated higher susceptibility to MALE and MACE in prediabetes compared to normoglycemic counterparts, notwithstanding lower HbA1c levels. Complications may manifest at an earlier prediabetes trajectory. Intensive lifestyle modification may improve the prognosis of severe prediabetes.

## Introduction

Diabetes mellitus (DM) is a global health concern projected to increase by 50% in the next 25 years [1]. DM leads to microvascular and macrovascular complications, impacting organs and significantly reducing patients’ quality of life [2–4]. In individuals with DM, hyperglycemia-induced lipid changes contribute to peripheral arterial occlusive disease, destabilizing plaques and resulting in substantial cardiovascular events [5, 6]. The EUCLID study highlights the significant impact of DM on PAD outcomes, revealing a 14.2% increase in major cardiovascular events for every 1% rise in HbA1c levels. Concurrent PAD and coronary heart disease amplify the risk of major adverse cardiovascular events by 50% compared to PAD alone [7, 8].

Prediabetes, an intermediate stage between normal blood sugar levels and a diabetes diagnosis, is of growing concern. Globally, the incidence of type 2 diabetes is anticipated to surpass 600 million individuals by 2045, with a corresponding number having prediabetes [9]. As defined by the American Diabetes Association (ADA), prediabetes is characterized by elevated blood glucose levels (fasting plasma glucose 100–125 mg/dl and/or 2-hour plasma glucose 140–199 mg/dl after an oral glucose challenge, and/or hemoglobin A1C 5.7–6.4%) [10]. The early stage of diabetes development involves a compensatory phase marked by insulin resistance, increased insulin production, and an enlarged β-cell mass. Subsequently, there is a sustained adaptation phase where β-cells fail to fully compensate for insulin resistance, leading to inadequate glucose regulation. This phase typically begins with normal fasting and postload glucose levels, possibly with reduced acute insulin production [11, 12]. Prediabetes elevates the risk of advancing to overt diabetes, with an estimated yearly progression rate spanning 5–15% [13]. Recent studies have established prediabetes as an independent risk factor for cardiovascular disease and a higher risk of adverse cardiovascular events [14, 15].

While the association between DM and PAD is established, the role of prediabetes remains incompletely understood. Clarifying the prognostic implications and a precise definition may guide interventions for improved patient outcomes. The study aims to investigate the relationship between prediabetes and both limb-related complications and cardiovascular disease.

## Methods

### Study population

This study conducted a longitudinal retrospective cohort analysis using electronic health records (EHRs) from patients aged 45 or older who received medical care at a tertiary medical facility in Taiwan between January 1, 2014, and December 31, 2019. The EHRs were obtained from the National Taiwan University Hospital Integrated Medical Database (NTUH-iMD) [16]. Prior research has established the consistent quality of medical data for generating real-world evidence [17]. The NTUH-iMD is a comprehensive data collection system that aggregates information from Taipei Main Hospital and its affiliated branches in at least four counties across Taiwan. These digitized EHRs have been accessible through online platforms since 2006 and contain a wide range of patient-related information, including demographics, diagnoses, physician prescriptions, laboratory results, interventions, medications, and examinations. Approval for this study was obtained from the Institutional Review Board of the National Taiwan University Hospital (NTUH-REC No.202007138RIND).

We excluded from the study individuals who did not undergo blood glucose testing or were diagnosed with overt diabetes based on the International Classification of Diseases (ICD) code, or who were treated with any antidiabetic medication throughout the duration of the study. We also excluded patients who had a previous history of PAD, critical limb ischemia, amputation, acute myocardial infarction, or stroke. In accordance with the 2023 guidelines established by the American Diabetes Association, prediabetes was defined as having a fasting plasma glucose (FPG) level of 100–125 mg/dL (5.6–6.9 mmol/L), a two-hour plasma glucose (PG) level of 140 − 199 mg/dL (7.8–11.0 mmol/L) during a 75 g oral glucose tolerance test (OGTT), or an HbA1c level falling within 5.7–6.4% (39–47 mmol/mol) [10]. Normal glucose regulation (NGR) is defined as having fasting plasma glucose levels below 100 mg/dL (5.6 mmol/L) and 2-hour plasma glucose levels below 140 mg/dL (7.8 mmol/L) during an oral glucose tolerance test (OGTT) [18].

We classified participants into four subclasses to facilitate a clearer comparison of the diabetes spectrum’s impact over an extended follow-up period. These classifications are based on the initial and last glucose test results without pharmacological intervention before the end of the study. “NGR with progress” denotes individuals who started with NGR but progressed to prediabetes or diabetes by the study’s conclusion. In contrast, “NGR without progress” describes those who consistently maintained NGR throughout the entire study, without transitioning to prediabetes or diabetes. “Prediabetes with progress” refers to individuals initially diagnosed with prediabetes who advanced to diabetes by the study’s conclusion. Conversely, “Prediabetes without progress” characterizes individuals who either remained in the prediabetic state or reverted to NGR.

We used EHR data to evaluate baseline characteristics, such as age, gender, body mass index (BMI), hypertension, hyperlipidemia, heart failure, coronary artery disease (CAD), chronic kidney disease, chronic pulmonary disease (COPD), and transient ischemic attack (TIA)/ischemic stroke. The index date was determined as the date of the first recorded glucose test, including for FPG, two-hour PG, and HbA1c levels. Conditions diagnosed anterior to the index date were categorized as etiologies using ICD codes. On or after the index date, we collected data on renal function, lipid profile (total cholesterol [TCHO], low-density lipoprotein [LDL], high-density lipoprotein, and triglycerides), and N-terminal-pro-B type natriuretic peptide [NT-pro-BNP]. The estimated glomerular filtration rate (eGFR) was computed with the Modification of Diet in Renal Disease equation.

In this study, the composite endpoint included major adverse limb events (MALE), which were defined as the initial diagnosis of either PAD or critical limb ischemia. Major adverse cardiovascular events (MACE) were defined as a combination of cardiovascular mortality, non-fatal myocardial infarction, and non-fatal ischemic stroke. A central committee determined the cause of death. We reviewed all available medical records until the last clinical visit or death, whichever came first.

### Statistical analysis

Categorical variables are reported as percentages, while continuous variables are reported as means with standard deviations. The Shapiro-Wilk test was used to assess normality in small samples of continuous variables. If non-normally distributed, we applied the Mann-Whitney U test for group comparisons. For large samples, assuming normal distribution, we compared groups using the Student’s t-test for continuous variables and the chi-square test for categorical variables. We calculated the incidence, age-standardized incidence, incidence rate, and incidence rate ratio of MALE. The proportional hazards hypothesis was confirmed using scaled Schoenfeld residuals and hazard ratio plots. Crude hazard ratios (cHRs) and adjusted hazard ratios (aHRs) with their corresponding 95% confidence intervals (CIs) were estimated employing multivariable Cox regression models. Model 1 was the rudimentary model, while model 2 was further adjusted for baseline characteristics, including age, sex (with women serving as the reference group), BMI, hypertension, hyperlipidemia, heart failure, CAD, COPD, and eGFR. For subgroup analyses, forest plots with aHRs, CIs, and *p*-values were employed. The cumulative incidence of MACE and MALE was estimated using the Kaplan–Meier method, and the significance of the difference between the incidence curves was determined using log-rank tests.

All statistical analyses were performed using R (version 4.1.2), SAS version 9.4 (SAS Institute Inc., Cary, NC, USA), and SPSS version 25.0 (SPSS Inc., Chicago, IL, USA). A *p*-value of less than 0.05 was considered statistically significant.

## Results

### Baseline characteristics

**Fig. 1:**
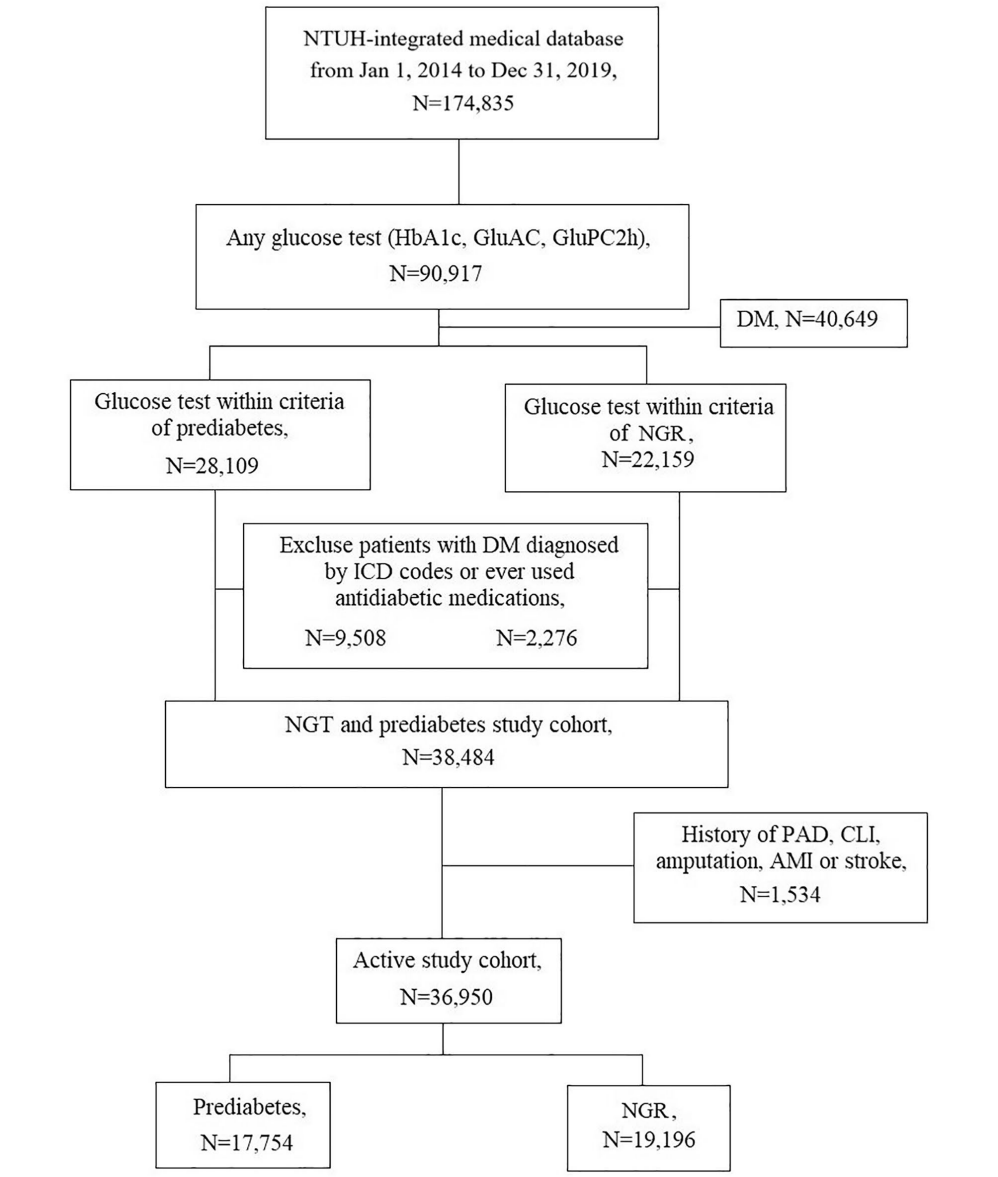
Study flowchart

Figure 1 illustrates the patient selection process for the cohort from 2014 to 2019. The initial cohort comprised 174,835 individuals, with 90,917 excluded due to a lack of glucose testing and an additional 40,649 excluded based on glucose levels indicative of DM. Further exclusions encompassed individuals with a prior ICD diagnosis of DM or a history of antidiabetic medication use. The subsequent refinement excluded 1,534 individuals who experienced the outcomes of interest, either MALE or MACE, before enrollment. The final analysis cohort consisted of 36,950 individuals. These subjects were then categorized into having normal glucose tolerance (NGR; 19,196 participants) or prediabetes (17,754 individuals).

Table 1 shows the clinical, biochemical, and anthropometric characteristics of the participants. Patients with prediabetes were older (65.2 ± 10.7 years vs. 62.9 ± 10.2 years, *p* < 0.001), more likely to be men (48.9% vs. 40.7%, *p* < 0.001), had higher BMI (25.1 ± 4.1 vs. 23.6 ± 3.8, *p* < 0.001), and had more comorbidities, including hypertension (27.6 vs. 21.2%, *p* < 0.001), hyperlipidemia (19.3% vs. 14.6%, *p* < 0.001), heart failure (0.7% vs. 0.5%, *p* = 0.029), atrial fibrillation (3.7% vs. 2.8%, *p* < 0.001), CAD (12.9% vs. 8.7%, *p* < 0.001), and COPD (3.7% vs. 3.0%, *p* = 0.011). On the other hand, patients with NGR had higher serum TCHO (189.1 ± 40.3 mg/dL vs. 186.1 ± 41.9 mg/dL, *p* < 0.001), HDL (53.6 ± 14.7 mg/dL vs. 49.1 ± 13.4 mg/dL, *p* < 0.001), and LDL levels (113.1 ± 32.5 mg/dL vs. 112.1 ± 33.9 mg/dL, *p* = 0.029), but had lower triglyceride levels (141.8 ± 96.4 mg/dL vs. 121.7 ± 82.6 mg/dL, *p* < 0.001). Patients with prediabetes had lower levels of NT-pro-BNP (2477.0 ± 6377.9 pg/ mL vs. 3364.0 ± 8082.0 pg/ mL, *p* = 0.177).

**Table 1:** Patients’ demographics

	NGR			Prediabetes			*p* value (normal vs. prediabetes)
	Overall N = 19,196	NGR with progress N = 3,672	NGR without progress N = 15,524	Overall N = 17,754	Prediabetes with progress N = 2,920	Prediabetes without progress N = 14,834	
Age, yr	62.9 ± 10.4	64.0 ± 10.4	62.7 ± 10.4	65.2 ± 10.2	64.8 ± 9.9	65.2 ± 10.2	**< 0.001**
Male	7,821 (40.7)	1,702 (46.4)	6,119 (39.4)	8,675 (48.9)	1,489 (51.0)	7,186 (48.4)	**< 0.001**
BMI, kg/m2	23.6 ± 3.8	24.52 ± 4.0	23.4 ± 3.7	25.1 ± 4.1	25.9 ± 4.3	24.9 ± 4.0	**< 0.001**
History of hypertension	4,076 (21.2)	1,107 (30.2)	2,969 (19.1)	4,902 (27.6)	993 (34.0)	3,909 (26.4)	**< 0.001**
History of hyperlipidemia	2,806 (14.6)	893 (24.3)	1,913 (12.3)	3,421 (19.3)	744 (25.5)	2,677 (18.1)	**< 0.001**
History of HF	104 (0.5)	25 (0.7)	79 (0.5)	128 (0.7)	17 (0.6)	111 (0.8)	**0.029**
History of CAD	1,663 (8.7)	523 (14.2)	1,140 (7.3)	2,290 (12.9)	458 (15.7)	1,832 (12.4)	**< 0.001**
History of CKD	359 (1.9)	77 (2.1)	282 (1.8)	296 (1.7)	40 (1.4)	256 (1.7)	0.140
History of Gout	198 (1.0)	31 (0.8)	167 (1.1)	235 (1.3)	33 (1.1)	202 (1.4)	**0.009**
History of COPD	582 (3.0)	116 (3.2)	466 (3.0)	622 (3.5)	108 (3.7)	514 (3.5)	**0.011**
History of AF	530 (2.8)	22 (0.6)	508 (3.3)	663 (3.7)	19 (0.7)	644 (4.3)	**< 0.001**
Baseline HbA1C, %	5.4 ± 0.2	5.4 ± 0.2	5.4 ± 0.2	5.9 ± 0.3	6.1 ± 0.3	5.9 ± 0.3	**< 0.001**
Baseline FG, mmol/L	90.1 ± 7.1	91.7 ± 7.3	89.7 ± 7.1	105.9 ± 9.4	110.5 ± 9.1	105.0 ± 9.2	**< 0.001**
eGFR, mL / min / 1.73m2	75.2 ± 26.2	75.1 ± 27.6	75.3 ± 25.9	75.0 ± 27.2	77.4 ± 29.3	74.5 ± 26.7	0.494
TG, mg/dL	121.7 ± 82.6	138.8 ± 96.1	116.9 ± 77.7	141.8 ± 96.4	157.9 ± 115.3	138.0 ± 91.0	**< 0.001**
TCHO, mg/dL	189.1 ± 40.2	185.9 ± 42.7	189.9 ± 39.5	186.1 ± 41.9	182.2 ± 42.8	187.0 ± 41.7	**< 0.001**
LDL, mg/dL	113.1 ± 32.5	112.7 ± 34.0	113.2 ± 32.0	112.1 ± 33.9	110.5 ± 34.8	112.5 ± 33.7	0.294
HDL, mg/dL	53.6 ± 14.7	49.7 ± 13.9	54.9 ± 14.7	49.1 ± 13.4	45.9 ± 11.9	49.9 ± 13.6	**< 0.001**
NT-proBNP, pg/ mL	3364.0 ± 8082.0	3652.5 ± 8514.1	3208.0 ± 7839.4	2477.0 ± 6377.9	3149.5 ± 7818.7	2291.3 ± 5908.7	**0.177**

* NGR, normal glucose regulation

Abbrev. BMI, body mass index; HF, heart failure; CAD, coronary artery disease; CKD, chronic kidney disease; COPD, chronic obstructive pulmonary disease; AF, atrial fibrillation; FG, fasting glucose; eGFR, estimated glomerular filtration rate; TCHO, total cholesterol; LDL, low-density lipoprotein; HDL, high-density lipoprotein; NTproBNP, N-terminal prohormone of brain natriuretic peptide

### Outcomes

Table 2 presents the cumulative incidence and incidence rates of MALE and MACE. The median duration of follow-up was 46.4 months. The cumulative incidence of MALE was 3.58%. After adjusting for age, the incidence of MALE was 2.86% in the NGR group and 3.22% in the prediabetes group. During a follow-up period of 131,783 person-years, the MALE and MACE incidence rates were 10.22 and 9.68 per 1,000 person-years, respectively. In the NGR group, the incidence rate of MALE was 9.53 per 1,000 person-years, while it was 10.80 per 1,000 person-years in the prediabetes group. The incidence rate of MACE in the NGR group was 7.56 per 1,000 person-years, while it was 11.99 per 1,000 person-years in the prediabetes group. The incidence rate ratio was 1.08 (95% CI: 0.93–1.26) for MALE and 1.20 (95% CI: 0.84–1.71) for MACE.

**Table 2:** Cumulative incidence and incidence rates of major adverse limb events (MALE) and major adverse cardiovascular events (MACE).

		N	Event	Incidence (%)	Age standardized incidence (%)	Incidence rate (per 1000 person-year)	Time at risk (person-year)	Median follow-up time (month)	Incidence rate ratio (IRR) and 95%CI
**MALE**									
NGR	Overall	19,196	646	3.37	2.86	9.53	67,756.65	46.43	0.95 (0.87–1.05)
	NGR with progress	3,672	156	4.25	3.46	10.38	15,030.08	56.17	1.04 (0.96–1.12)
	NGR without progress	15,524	490	3.16	2.71	9.29	52,726.57	43.97	0.93 (0.80–1.07)
Prediabetes	Overall	17,754	678	3.82	3.22	10.80	62,750.82	46.37	1.08 (0.93–1.26)
	Prediabetes with progress	2,920	133	4.55	3.69	11.15	11,925.41	57.35	1.12 (0.90–1.38)
	Prediabetes without progress	14,834	545	3.67	3.15	10.72	50,825.41	44.03	1.07 (0.94–1.23)
**MACE**									
NGR	Overall	19,196	519	2.70	2.27	7.56	68,634.90	47.10	0.76 (0.44–1.31)
	NGR with progress	3,672	160	4.36	3.68	10.59	15,101.72	56.60	1.06 (0.95–1.19)
	NGR without progress	15,524	359	2.31	1.90	6.71	53,533.18	44.57	0.67 (0.31–1.47)
Prediabetes	Overall	17,754	757	4.26	3.76	11.99	63,147.89	46.80	1.20 (0.84–1.71)
	Prediabetes with progress	2,920	161	5.51	4.92	13.45	11,966.73	57.67	1.35 (0.75–2.41)
	Prediabetes without progress	14,834	596	4.02	3.53	11.64	51,181.16	44.47	1.16 (0.86–1.57)

* NGR, normal glucose regulation

**Fig. 2:**
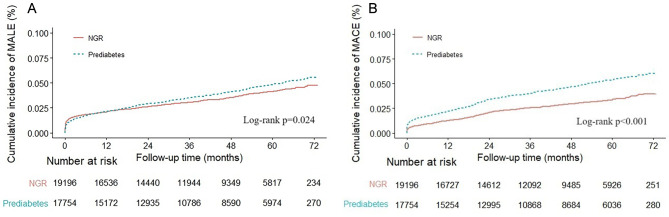
Kaplan–Meier analysis of (**A**) major adverse limb events (MALE) and (**B**) major adverse cardiovascular events (MACE).

Figure 2 presents the Kaplan-Meier analysis, revealing a significant increase in MALE and MACE in individuals with prediabetes. The log-rank test confirms statistical significance (*p* = 0.024 for MALE and *p* < 0.001 for MACE).

Tables 3 and 4 delineate subgroup analyses stratified risks associated with MALE and MACE concerning HbA1c levels, IFG, and IGT. In Table 3, the prediabetes group exhibited a heightened risk of MALE (aHR 1.26 [1.10–1.46], *p* = 0.001). Within this prediabetes group, both those without progress and those with progress demonstrated increased susceptibility to MALE (aHR 1.23 [1.06–1.42], *p* = 0.007;a HR 1.43 [1.15–1.77], *p* = 0.001, respectively) when compared to the reference group of individuals with NGR without progress. The prediabetes categories with HbA1c levels between 5 and 5.5%, 5.5-6.0%, and 6.0-6.5% were diagnosed based on IFG or IGT. Individuals diagnosed with prediabetes based on IFG or IGT showed a significant association with increased MALE (cHR 1.17 [1.02–1.35], *p* = 0.023). However, the significance attenuated following adjustments for confounding factors (aHR 1.18 [1.00-1.39], *p* = 0.053). Individuals with HbA1c levels between 5 and 5.5% showed a non-significant association with MALE (cHR 1.46 [0.86–2.49], *p* = 0.160), which became significant after adjusting for confounders (aHR 1.78 [1.04–3.04], *p* = 0.036). For those with HbA1c levels between 5.5 and 6.0%, the adjusted hazard ratio was 1.29 (95% CI 1.06–1.58, *p* = 0.012), and for those with HbA1c levels between 6.0 and 6.5%, the adjusted hazard ratio was 1.39 (95% CI 1.14–1.70, *p* < 0.001).

**Table 3:** Multivariable Cox regression analysis of differences in MALE between individuals with NGR and prediabetes

	N	Event no.	Incidence rate (%)	Incidence (person-year)	Model 1				Model 2			
					HR	95% C.I.		*p* value	HR	95% C.I.		*p* value
						lower	upper			lower	upper	
NGR without progress	15,524	490	0.93	52,726.57	Ref.				Ref.			
NGR with progress	3,672	156	1.04	15,030.08	1.20	1.00	1.43	0.053	**1.45**	1.19	1.77	**< 0.001**
Prediabetes without progress	14,834	545	1.07	50,825.41	**1.16**	1.02	1.31	**0.020**	**1.23**	1.06	1.42	**0.007**
Prediabetes with progress	2,920	133	1.12	11,925.41	**1.28**	1.06	1.56	**0.011**	**1.43**	1.15	1.77	**0.001**
Prediabetes Overall	17,754	678	1.08	62,750.82	**1.18**	1.05	1.32	**0.006**	**1.26**	1.10	1.46	**0.001**
- HbA1c 3.5-5.0%	41	0	0.00	122.03	-				-			
- HbA1c 5.0-5.5%	329	14	1.46	959.59	1.46	0.86	2.49	0.160	**1.78**	1.04	3.04	**0.036**
- HbA1c 5.5-6.0%	4,346	157	1.03	15,271.34	1.12	0.93	1.34	0.223	**1.29**	1.06	1.58	**0.012**
- HbA1c 6.0-6.5%	4,001	165	1.12	14,737.09	**1.24**	1.04	1.48	**0.002**	**1.39**	1.14	1.70	**< 0.001**
- IFG or IGT	9,037	342	1.08	31,660.76	**1.17**	1.02	1.35	**0.023**	1.18	1.00	1.39	0.053
- IFG and IGT	120	3	0.62	482.82	0.71	0.23	2.21	0.553	1.02	0.33	3.17	0.978
- isolated IFG	8,971	340	1.08	31,447.77	1.18	1.02	1.35	0.022	1.18	1.00	1.39	0.054
- isolated IGT	186	5	0.72	695.80	0.80	0.33	1.93	0.620	1.07	0.44	2.60	0.875

* NGR without progress as reference

* Model 1: Crude; Model 2: Adjust age, sex, BMI, hypertension, hyperlipidemia, HF, gout, CAD, AF, eGFR

**Table 4:** Multivariable Cox regression analysis of differences in MACE between individuals with NGR and prediabetes

	N	Event no.	Incidence rate (%)	Incidence (person-year)	Model 1				Model 2			
					HR	95% C.I.		*p* value	HR	95% C.I.		*p* value
						lower	upper			lower	upper	
NGR without progress	15,524	359	0.67	53,533.18	Ref.				Ref.			
NFR with progress	3,672	160	1.06	15,101.72	**1.67**	1.39	2.02	**< 0.001**	**1.54**	1.27	1.88	**< 0.001**
Prediabetes without progress	14,834	596	1.16	51,181.16	**1.74**	1.53	1.98	**< 0.001**	**1.37**	1.19	1.59	**< 0.001**
Prediabetes with progress	2,920	161	1.35	11,966.73	**2.13**	1.77	2.56	**< 0.001**	**1.86**	1.52	2.28	**< 0.001**
Prediabetes Overall	17,754	757	1.20	63,147.89	**1.81**	1.60	2.05	**< 0.001**	**1.46**	1.27	1.67	**< 0.001**
- HbA1c 3.5-5.0%	41	1	0.82	122.01	1.14	0.16	8.10	0.898	0.79	0.11	5.60	0.810
- HbA1c 5.0-5.5%	329	13	1.33	975.63	**1.88**	1.08	3.28	**0.025**	1.52	0.87	2.66	0.138
- HbA1c 5.5-6.0%	4,346	217	1.42	15,250.41	**2.14**	1.81	2.54	**< 0.001**	**1.67**	1.39	2.01	**< 0.001**
- HbA1c 6.0-6.5%	4,001	262	1.79	14,626.20	**2.74**	2.33	3.21	**< 0.001**	**2.10**	1.76	2.51	**< 0.001**
- IFG or IGT	9,037	264	0.82	32,173.64	**1.24**	1.06	1.45	**0.008**	1.06	0.89	1.26	0.499
- IFG and IGT	120	5	1.04	481.32	1.64	0.68	3.96	0.274	1.42	0.53	3.83	0.484
- isolated IFG	8,971	259	0.81	31,963.71	**1.22**	1.04	1.44	**0.014**	1.05	0.89	1.25	0.565
- isolated IGT	186	10	1.45	691.25	**2.23**	1.19	4.18	**0.012**	1.72	0.85	3.48	0.130

* NGR without progress as referencence

* Model 1: Crude; Model 2: Adjust age, sex, BMI, hypertension, hyperlipidemia, HF, gout, CAD, AF, eGFR

In Table 4, prediabetes significantly increased the risk of MACE (aHR, 1.46 [1.27–1.67], *p* < 0.001). Furthermore, the risk of MACE increased earlier in individuals with prediabetes when HbA1c levels exceeded 5.5% (aHR 1.67 [1.39–2.01], *p* < 0.001 for HbA1c 5.5–6.0%; aHR 2.10 [1.76–2.51], *p* < 0.001 for HbA1c 6.0–6.5%). Individuals with isolated IFG were at a significantly higher risk of experiencing MACE (cHR 1.22 [1.04–1.44], *p* = 0.014) but the significance of this association diminished after confounding adjustment (aHR 1.05 [0.89–1.25], *p* = 0.565).Similarly, those prediabetes diagnosed by isolated IGT group showed the trend of increased MACE (cHR 2.23 [1.19–4.18], *p* = 0.012) but less significant after adjustment (aHR 1.72 [0.85–3.48], *p* = 0.130). Those with concomitant IFG and IGT also did not yield a higher risk of MACE (aHR 1.42 [0.53–3.83], *p* = 0.484).

**Fig. 3:**
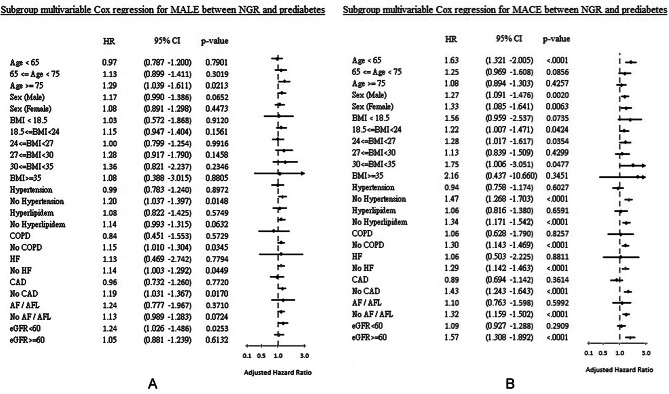
Subgroup analysis of (**A**) major adverse limb events (MALE) and (**B**) major adverse cardiovascular events (MACE).

In the prediabetes group, older age, male gender, a higher BMI, and a history of heart failure or atrial fibrillation were associated with both MALE and MACE. Figure 3 shows forest plots of HRs derived from the Cox model for subgroup analyses (NGR as reference).

## Discussion

In this retrospective observational study, we underscore the elevated risk of MACE and MALE in individuals with prediabetes when compared to those with NGR. This is the first study to investigate the potential association between prediabetes and PAD with a substantial sample size and regular longitudinal surveillance.

The annual conversion rate from prediabetes to diabetes generally ranges from 5 to 10%, resulting in around 70% of persons with prediabetes progressing to diabetes during their lifespans. The risk is significantly increased in those who are overweight or obese [19]. A meta-analysis has reported cumulative incidence rates of type 2 diabetes stemming from prediabetes over a four-year period, which vary between 14% and 27% [20]. In our study, around 80% of the participants sustained their blood glucose levels within the prediabetes range. Our results indicate that around 16% of individuals diagnosed with prediabetes progressed to diabetes within the designated follow-up period. While our observed outcomes align with previously reported cumulative incidence rates of prediabetes evolving into diabetes, it is plausible that our study has underestimated this transition. Nevertheless, a considerable proportion of patients remains in the prediabetes stage, concealing latent risks related to various health factors.

The continuum of progression from NGR to diabetes can be observed through longitudinal investigations that involve frequent monitoring of glucose levels, insulin sensitivity, and insulin production. The trajectories of fasting and postload glucose levels, as well as the trajectories of Homeostatic Model Assessment (HOMA) evaluations of insulin sensitivity and insulin secretion (β-cell function) leading up to the onset of type 2 diabetes have been elucidated in the British Whitehall II cohort [11, 21]. In individuals who later developed diabetes, elevated glucose levels were evident from the outset of the follow-up, up to 13 years before formal diagnosis. Prediabetes is of clinical importance as it significantly heightens the risk of diabetes development, associated complications, and related health issues compared to individuals with normal glucose levels. Those progressing from prediabetes to diabetes represent a high-risk subgroup [22, 23]. Beyond glycemic management, various comorbidities interact, and it is imperative to recognize the substantial diversity in risk profiles among the prediabetes population [24].

Prediabetes has been associated with a spectrum of health conditions, including cardiovascular disease, non-alcoholic fatty liver disease, neuropathy, chronic renal disease, cancer, dementia, and overall mortality [25, 26]. Previous research has indicated that while some individuals progress to diabetes, a more common occurrence is the regression to normoglycemia [27]. In our investigation, we refrained from quantifying the occurrences of a return to baseline blood glucose levels. Instead, we examined the effects observed in individuals who had experienced the prediabetes stage, regardless of whether they subsequently reverted to a normoglycemic state. The demographic characteristics of individuals diagnosed with high-risk prediabetes included several distinctive features, such as advanced age, male gender, elevated BMI, and a previous history of heart failure.

A multitude of putative mechanisms correlated with prediabetes to adverse outcomes. Even minor elevations in HbA1c levels can signify impaired glycemic control and insulin resistance. Prolonged hyperglycemia has the potential to rapidly increase inflammation, induce oxidative stress, worsen endothelial dysfunction, enhance foam cell formation, and stimulate smooth muscle proliferation. In combination, these fundamental pathological alterations can contribute to the development of atherosclerotic plaques within arterial walls, foster abnormal blood clotting, and initiate vascular remodeling. Consequently, these features ultimately contribute to an unfavorable prognosis in vascular diseases [28, 29]. Prior studies have indicated an association between prediabetes and an increased risk of heart failure. Within our cohort, despite an initial presentation of lower NTproBNP levels among prediabetic individuals, this encompassed cohorts that transitioned to NGR over the study period. In addition, the NGT group included individuals who subsequently progressed to prediabetes or diabetes. The evaluation of NTproBNP, particularly during symptomatic episodes, may introduce selection bias. In our subsequent analyses, the subgroup within the prediabetes cohort characterized by elevated NTproBNP levels consistently elevated risk of HF hospitalization, corroborating earlier research findings [30]. The prevalence of quantitatively assessed PAD, an initial clinical marker of atherosclerosis with a strong association with cardiovascular mortality, is significant in patients diagnosed with type 2 diabetes [31]. It is plausible that PAD may begin at an earlier stage than initially expected [32]. In the subgroup analysis confined to NGR without progress, we observed a significant escalation in MALE, even in the presence of lower HbA1c levels. Remarkably, the discernible impact of IFG or IGT persists. This underscores the pivotal role of stringent blood glucose control, emphasizing its substantial contribution to the reduction of MALE incidence.

The Atherosclerosis Risk in Communities (ARIC) Study uncovered substantial hazard associated with diagnosed diabetes, coronary heart disease, stroke, and all-cause mortality across various glycated hemoglobin levels. A distinctive J-shaped curve was evident in the connection between glycated hemoglobin and all-cause mortality, and this relationship remained statistically significant even after accounting for baseline fasting plasma glucose levels. Individuals with a glycated hemoglobin level of 6.0% or higher displayed an increased susceptibility to the development of diabetes, cardiovascular diseases, and stroke [33]. Furthermore, the link between glycated hemoglobin levels and overall mortality demonstrated a discernible trend [34]. In a systematic investigation that compared the diagnostic accuracy of various criteria with HbA1c, the significance of threshold implications for diagnosing specific conditions became evident. An HbA1c value of 6.0% consistently emerged as a robust predictor, indicating an increased hazard ratio concerning MACE [35]. Our study corroborates these observations and shows an earlier elevated risk of MACE at an HbA1c value of 5.5% in the Asian population.

Researchers have employed data-driven methodologies to classify DM metabolic phenotypes based on cardiovascular risk and BMI stratification [36, 37]. Challenges include the limited availability of OGTT data and variations in the prevalence of insulin resistance across different population subsets. Despite the shared feature of reduced early-stage insulin secretion in both IFG and IGT, epidemiological research indicates that these conditions follow distinct metabolic pathways and should be considered as separate populations with limited overlapping characteristics. Individuals with IGT exhibit substantial muscle insulin resistance, along with relatively modest hepatic insulin resistance and decreased late-phase insulin production [21, 38]. In contrast, individuals with IFG experience severe hepatic insulin resistance while maintaining normal or near-normal muscle insulin sensitivity.

Prediabetes is a highly heterogeneous metabolic state, characterized by diverse pathogenesis and disease prediction. Within Asian populations, distinct clusters might emerge, potentially associating HbA1c with PAD in one cluster and IFG in another, both suggesting increased MACE risk. However, thorough research is necessary to comprehensively explore these cardiometabolic risk clusters in prediabetes. Better understanding and precise phenotyping of prediabetes can enhance disease risk assessment. Additionally, the utilization of Mendelian randomization can help explore the causal association between genetic variations and prediabetes [39]. This study emphasizes the importance of managing HbA1c alongside addressing IFG to prevent MALE and MACE.

## Limitations

This study had several limitations. First, as it was a retrospective cohort study, surveillance bias and limited causal inference may have been present. Second, the oral glucose tolerance test was not routinely administered to the majority of individuals, resulting in an underestimation of prediabetes as defined by the IGT. However, HbA1c has been demonstrated to be a more precise indicator. Third, we did not investigate dynamic processes such as BMI fluctuation or lifestyle modification interventions. The occurrence of MACE and MALE may be influenced to some extent by adverse effects or treatments. However, because our population sample size was sufficiently large and we conducted regular follow-up at the medical center, the effect might have been diminished. Finally, it is crucial to recognize that our method of identifying changes between two time points may not capture every fluctuation in glucose levels throughout the entire duration. Variations in the frequency of glucose monitoring have the potential to introduce selection bias, thereby impacting the overall generalizability of the study.

## Conclusion

Our investigation substantiates an increased susceptibility to MALE and MACE in individuals with prediabetes compared to those with normoglycemia, notwithstanding lower HbA1c levels. The onset of complications appears to manifest at an earlier prediabetes trajectory. Intensive lifestyle modification may improve the prognosis of prediabetes patients at high risk.

## Data Availability

The datasets used in this study are available only at the National Taiwan University Hospital. The R programs (codes) used in this study are available from the corresponding author upon request.
